# Are JAKis more effective among elderly patients with RA, smokers and those with higher cardiovascular risk? A comparative effectiveness study of b/tsDMARDs in Sweden

**DOI:** 10.1136/rmdopen-2023-003648

**Published:** 2023-12-26

**Authors:** Hannah Bower, Thomas Frisell, Daniela di Giuseppe, Benedicte Delcoigne, Ulf Lindström, Carl Turesson, Katerina Chatzidionysiou, Elisabet Lindqvist, Ann Knight, Helena Forsblad-d'Elia, Johan Askling

**Affiliations:** 1 Clinical Epidemiology Division, Department of Medicine Solna, Karolinska Institutet, Stockholm, Sweden; 2 Department of Rheumatology and Inflammation Research, Institute of Medicine, Sahlgrenska Academy at the University of Gothenburg, Gothenburg, Sweden; 3 Rheumatology, Department of Clinical Science Malmö, Lund University, Skåne University Hospital, Malmö, Sweden; 4 Rheumatology, Theme Inflammation and Ageing, Karolinska University Hospital, Stockholm, Sweden; 5 Section of Rheumatology, Department of Clinical Sciences Lund, Lund University, Skåne University Hospital, Lund, Sweden; 6 Rheumatology, Department of Medical Sciences, Uppsala University, Uppsala, Sweden

**Keywords:** Arthritis, Rheumatoid, Biological Therapy, Epidemiology, Tumor Necrosis Factor Inhibitors

## Abstract

**Objectives:**

To investigate whether the relative effectiveness of janus kinase inhibitors (JAKis) versus tumour necrosis factor inhibitors (TNFi) or other biological disease-modifying antirheumatic drugs in rheumatoid arthritis differ by the presence or absence of risk factors for cardiovascular (CV) disease, age, sex and smoking.

**Methods:**

Through Swedish registers, we identified 13 493 individuals with 3166 JAKi, 5575 non-TNFi and 11 286 TNFi treatment initiations 2016–2022. All lines of therapy were included, with the majority in second line or higher. Treatment response was defined as the proportion reaching European Alliance of Associations for Rheumatology (EULAR) good response and Clinical Disease Activity Index (CDAI) remission, respectively, within 6 months. Crude percentage point differences in these proportions (JAKis, and non-TNFis, vs TNFis) overall and by risk factors were observed, and adjusted for confounders using linear regression models. Predicted probabilities of response and remission were estimated from adjusted Poisson models, and presented across CV risk and age.

**Results:**

Overall, adjusted percentage point differences indicated higher response (+5.0%, 95% CI 2.2% to 7.9%) and remission (+5.8%, 95% CI 3.2% to 8.5%) with JAKis versus TNFis. The adjusted percentage point differences for response in those above 65, at elevated CV risk, and smokers were +5.9% (95% CI 2.7% to 9.0%), +8.3% (95% CI 5.3% to 11.4%) and +6.0% (95% CI 3.3% to 8.7%), respectively. The corresponding estimates for remission were +8.0% (95% CI 5.3% to 10.8%), +5.6% (95% CI 3.0% to 8.2%) and +7.6% (95% CI 5.5% to 9.7%).

**Conclusions:**

As used in clinical practice, response and remission at 6 months with JAKis are higher than with TNFi. Among patients with risk factors of concern, effectiveness is similar or numerically further increased. For individualised benefit-to-risk ratios to guide treatment choice, safety and effectiveness in specific patient segments should be considered.

**WHAT IS ALREADY KNOWN ON THIS TOPIC**

- Evaluation of the benefit-to-risk ratio for individual drugs requires information on risks as well as benefits, overall and in defined patient segments.
- Based on the results of the Oral Rheumatoid Arthritis Trial (ORAL) surveillance safety trial of the janus kinase inhibitor (JAKi) tofacitinib, regulatory agencies have issued warnings and precautions regarding the use of the class of JAKis in certain patient segments: individuals above 65 years of age, smokers, and those at elevated risk of cardiovascular events or cancer.
- Effectiveness of JAKis has been reported to be on par or even superior to tumour necrosis factor inhibitors (TNFi), but whether this holds true also in those patient segments where risks (relative to TNFi) are increased remains unclear, but of critical importance for individualised benefit-to-risk ratios.

**WHAT THIS STUDY ADDS**

- We demonstrate that as used in clinical practice against rheumatoid arthritis (RA), the effectiveness of JAKi in terms of EULAR good response and CDAI remission is superior to TNFi, overall, also or even especially so in those patient with RA segments where safety concerns have been raised (here: elderly, smokers, and patients at increased cardiovascular risk).

**HOW THIS STUDY MIGHT AFFECT RESEARCH, PRACTICE OR POLICY**

- The observation that the relative effectiveness (response) of JAKis was similar or numerically increased in those patient segments where the safety concerns with JAKi are the biggest should help guide the choice of treatment in clinical practice.

## Introduction

Data from the ORAL surveillance trial of the janus kinase inhibitor (JAKi) tofacitinib has resulted in safety concerns, specifically for serious infections, venous thrombotic events, cardiovascular disease (CVD) and cancer, extrapolated to the entire class of JAKi approved for rheumatoid arthritis (RA) despite the ORAL surveillance trial being limited to a specific cardiovascular (CV) risk enriched trial population.^1^ The European Medicines Agency currently advises that among patients above 65 years of age, in smokers and in those at elevated CV or cancer risk JAKis only be used where no other suitable treatment alternatives are available.^2^ Observational studies in different settings have (but to varying degrees) been able to replicate results regarding safety outcomes seen in the ORAL surveillance trial.^3–8^

When selecting treatment for individuals with RA, both safety and effectiveness are important aspects to consider and should be evaluated in the format of a risk–benefit assessment tailored to the individual to be treated, as highlighted, for example, by the focus on both effectiveness and safety in the European Alliance of Associations for Rheumatology (EULAR) 2022 recommendations for the management of RA.^9^ Such assessments are especially important in RA where many treatment options are available. Therefore, although the debate around the use of JAKis in RA tends to focus on safety, in specific patient segments, their benefit-to-risk ratio should be informed also by their relative effectiveness in the very same patient segments.

Regarding effectiveness overall, studies have shown JAKis to be non-inferior, or even superior, to tumour necrosis factor inhibitors (TNFis) both in clinical trials and in observational settings.^10–14^ After recent evidence of differences in safety in distinct patient segments, it thus becomes relevant to also ask whether there are differences in the effectiveness of JAKis in the very same specific patient segments. However, to our knowledge, there has been no published assessment of relative effectiveness within defined patient subpopulations, here: specifically, in those patient groups where the safety concerns with JAKis may be the highest.

We therefore aimed to determine whether the relative effectiveness of JAKis versus biological disease-modifying antirheumatic drugs (bDMARDs) in RA differ by the presence or absence of factors that currently constitute reason for safety concerns, that is, age, smoking and elevated CVD risk. As a secondary objective, we aimed to put these results in perspective by quantifying the relative effectiveness also of non-TNFi bDMARDs versus TNFi, since these bDMARDs are an integral part of the treatment landscape.

## Methods

### Study design and data sources

We conducted a nationwide register-based cohort study using prospectively collected and linked data from Swedish registers. Information on patients with RA was obtained from the Swedish Rheumatology Quality register (SRQ) as part of a register linkage (Anti-Rheumatic Therapies in Sweden).^15^ This linkage contains data from the National Patient Register (NPR), the Prescribed Drug Register, the Cause of Death Register, the Swedish Total Population Register, the Cancer Register and Longitudinal integrated database for health insurance and labour market studies (LISA).

### Study population and treatment exposures

Patients above 18 years of age with RA and with a recorded biological or targeted synthetic disease-modifying antirheumatic drug (b/tsDMARD) treatment initiation were identified in the SRQ between 2016 and July 2022. Three treatment cohorts were created including the first treatment episode per molecule per individual: (1) TNFis (adalimumab, certolizumab pegol, etanercept, golimumab, infliximab), (2) non-TNFis bDMARDs (rituximab, abatacept, tocilizumab, sarilumab), and (3) JAKis (baricitinib, tofacitinib, upadacitinib, filgotinib). One individual could thus contribute to more than one treatment cohort (eg, a patient starting a TNFi, then a non-TNFi, then a JAKi would contribute to all three cohorts), more than once to the same treatment cohort (eg, a patient starting etanercept, later treated with infliximab), but only once with each molecule (eg, a patient first starting baricitinib, then switched to abatacept, then restarting baricitinib would only contribute the first of the two baricitinib treatments). Treatments that were stopped but restarted within 90 days were regarded as one consecutive treatment episode. Switching between an originator product and a biosimilar was not considered a treatment change. All lines of therapy were included, with the majority in second line or higher.

### Outcomes

Information on EULAR good response and CDAI remission outcomes was obtained via rheumatology visits between 60 and 180 days after treatment initiation. EULAR good response was defined as a DAS28 value≤3.2 at a rheumatology visit within this window, plus the requirement of a reduction in DAS28 value of 1.2 from baseline value, and conditioned on the patients remaining on drug at least up until that visit. Remission was defined as a CDAI values of ≤2.8 at a rheumatology visit within this window, also conditioned on drug survival up until that time-point.

Individuals who died or emigrated before 60 days after the treatment initiation in question were excluded (n=7). Individuals who immigrated to Sweden in the 10 years prior to the treatment initiation in question were not included.

Treatment episodes were imputed as non-response/non-remission when treatment was discontinued (for any reason other than pregnancy and inactive disease) prior to reaching the outcome within 180 days after initiation. Patients who discontinued due to pregnancy during this window were treated as having missing outcomes, whereas those who discontinued due to inactive disease were treated as good responders/reaching remission; online supplemental table S2 presents the number of non-response/non-remission imputations.

10.1136/rmdopen-2023-003648.supp1 Supplementary data

### Covariates

Information on age, sex and disease-related factors at baseline (seropositive RA, health assessment questionnaire (HAQ), Visual Analogue Scale (VAS) pain, disease duration, number of previous b/tsDMARDs), and smoking were obtained from the SRQ. The Prescribed Drug Register was used to define drug dispensations around treatment initiation (methotrexate, other csDMARDs, antidiabetics, antihypertensives, lipid lowering drugs, prednisolone use). Diagnoses of comorbidities were defined using the NPR (history of: joint surgery, diabetes, hyperlipidaemia, hypertension, myocardial infarction and stroke). Highest education at treatment initiation was obtained from LISA, and information on whether the individual was born in Sweden was obtained from the Total Population Register. Detailed baseline covariate definitions are available in online supplemental table S1.

CV risk was defined in two ways, and used in parallel: (1) as a dichotomous variable for the presence of ≥1 CV risk factor (hypertension, lipid-lowering drugs, diabetes, CVD, family history of CVD, smoker, see online supplemental table S1) plus C-reactive protein (CRP)≥3 and Disease Activity Score on 28 joints (DAS28CRP)≥2.9, in an attempt to select a patient population similar to that in the ORAL surveillance trial, and (2) as a continuous variable according to the Expanded Risk Score in RA (ERS-RA) which quantifies CV risk on a scale of 0–100 accounting for age, sex, smoking, CV risk factors (diabetes, hyperlipidaemia, hypertension), CDAI, HAQ, prednisolone use and RA duration,^16 17^ see online supplemental table S1 for details.

### Statistical methods

Summary statistics of baseline characteristics were calculated by treatment cohort. The number and percentage of each outcome were calculated over treatment cohort, and within subgroups by sex, age (<65, ≥65 years), CV risk (Y/N) and smoking (ever/never).

Crude percentage point differences in the proportions reaching each outcome were calculated comparing JAKis, and non-TNFI bDMARDs, to TNFis. Adjusted percentage point differences were estimated from linear regression models^18^ adjusting for age, sex, presence versus absence of CV risk, smoking, line of therapy, baseline DAS28CRP (for response outcome) and baseline CDAI (for remission outcome) seropositive RA, HAQ, VAS pain, RA duration, history of joint surgery, concomitant methotrexate and other csDMARD use, prednisolone use in previous year, year of treatment initiation, country of birth (Sweden or other) and education. A broad range of covariates were used in adjusted statistical analyses to accommodate the potential for confounding by indication, while ensuring covariates were not collinear and not on the causal pathway. Separate models were fitted for JAKis versus TNFis, and non-TNFis versus TNFis. Analyses were also performed by line of therapy by inclusion of interaction terms between cohort and line of therapy.

In parallel, adjusted linear regression models were fitted per outcome, modelling CV risk according to ERS-RA score (continuous) and age continuously using restricted cubic splines with three degrees of freedom, and allowing for effect-modification by treatment; risk differences were plotted across these continuous variables by treatment cohort. Similarly, adjusted Poisson models were fitted in order to present smooth estimates of the predicted risk ratio of each outcome, over CV ERS-RA score and age, by treatment initiation. Predicted risk estimates were presented for a specific covariate pattern defined by the median values in the datasets, representing the ‘typical’ patient with RA.

All models used robust standard errors to account for the dependence between observations, since the main analysis allowed one individual to contribute more than one treatment episode.

Multiple imputation by chained equations^19 20^ was performed for all variables with missing values, including both baseline covariates and outcomes: EULAR good response (56% missing), CDAI remission (47% missing), VAS pain, HAQ, CDAI, DAS28CRP, CV risk score (both Y/N, and continuous CV ERS-RA score), education, smoking, seropositive RA, RA duration. Missing values were assumed to be missing at random. All variables were imputed using the same functional form as in the analysis model. Conditional imputation was performed for the outcomes, conditioning on those individuals without non-responder imputation. Predictive mean matching was used for continuous variables, with k=5 nearest neighbour matching. Augmented logistic regression was implemented for imputation of binary variables. Fifty (50) imputations with 30 burn-in iterations were performed and estimates combined using Rubins Rules.^21^ The mi package in Stata V.16 was used to perform multiple imputation. A complete case analysis was performed as a sensitivity analysis.

### Sensitivity analyses

We performed sensitivity analyses to explore the extent to which the study design affected the observed results, specifically we: (1) excluded all treatment episodes in patients with no previous b/tsDMARD use (as JAKi has not been a first-line option for b/tsDMARD), and (2) restricted analyses to those individuals who initiated a TNFi, a non-TNFi, or a JAKi as second ever b/tsDMARD following a TNFi as first ever b/tsDMARD. An additional sensitivity analysis was performed including treatment initiations to August 2019, to avoid issues due to changes in treatment and care of RA patients during the COVID pandemic.

### Patient and public involvement

Patients or the public were not involved in the study design, conduct, reporting, or dissemination plans of our research, but contributed data through patient-reported data in the SRQ.

## Results

We identified 13 493 individuals with RA contributing to 3166 JAKi, 5575 non-TNFi bDMARD and 11 286 TNFi treatment initiations between 2016 and 2022 in Sweden. The majority of JAKi initiations were baricitinib (n=1928), with fewer initiations of tofacitinib (n=459), upadacitinib (n=755) and filgotinib (n=24). Patient characteristics across the treatment cohorts differed, most noticeably for RA duration (median 13.5 years, 12.0 years and 7.4 years for JAKis, non-TNFi bDMARDs and TNFis, respectively), number of previous b/tsDMARD (50%, 31% and 6% with 3 or more, for JAKis, non-TNFi bDMARDs and TNFis, respectively). The median age at treatment initiation was 59–63 years; the proportion female was 77%–82% across the cohorts. 57%–61% were previous or current smokers, and 37%–45% of the 3 cohorts were at elevated CV risk as defined in online supplemental table S1. Full characteristics are presented in online supplemental table S3. After multiple imputation, distributions of variables with missing information remained similar to the original dataset (online supplemental table S4).

### Effectiveness overall

**Figure 1:**
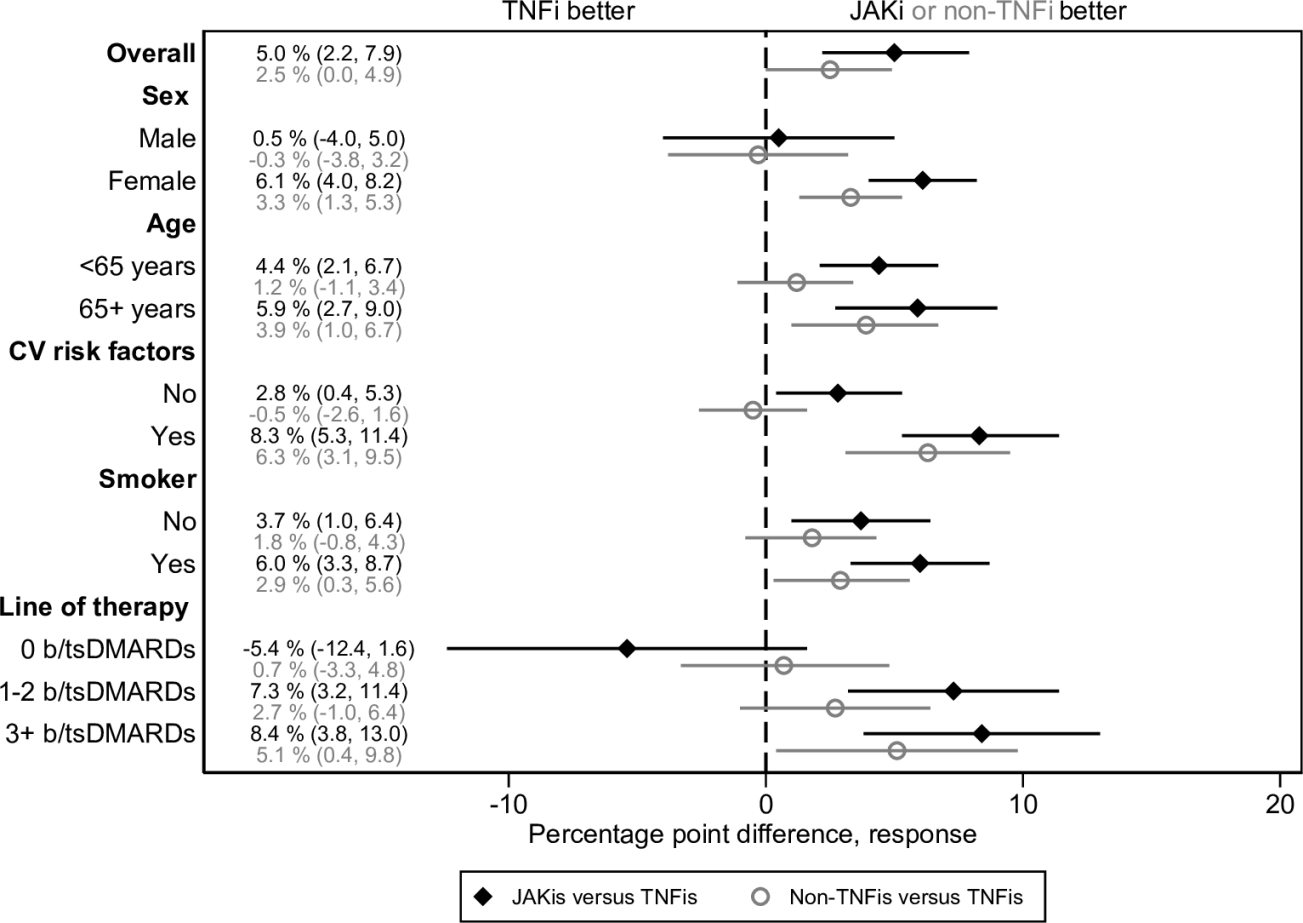
Percentage point differences for JAKis and non-TNFis (vs TNFi) of reaching EULAR good response at 6 months, presented overall, by sex, cardiovascular (CV) risk groups, and line of therapy. b/tsDMARD, biological or targeted synthetic disease-modifying antirheumatic drug; JAKi, janus kinase inhibitor; TNFi, tumour necrosis factor inhibitor.

Overall, 1074 (34%), 1891 (34%) and 4228 (38 %) JAKi, non-TNFi and TNFi initiations achieved EULAR good response at 6 months, see table 1. Crude percentage point differences indicated a better response in TNFi versus JAKi or non-TNFi, but when adjusting for covariates, the proportion reaching EULAR good response was instead the highest for JAKi; the fully-adjusted percentage point difference for JAKi versus TNFi was +5.0% (95% CI 2.2% to 7.9%) and +2.5% (95% CI 0.0% to 4.9%) for non-TNFi versus TNFi, see figure 1.

**Figure 3:**
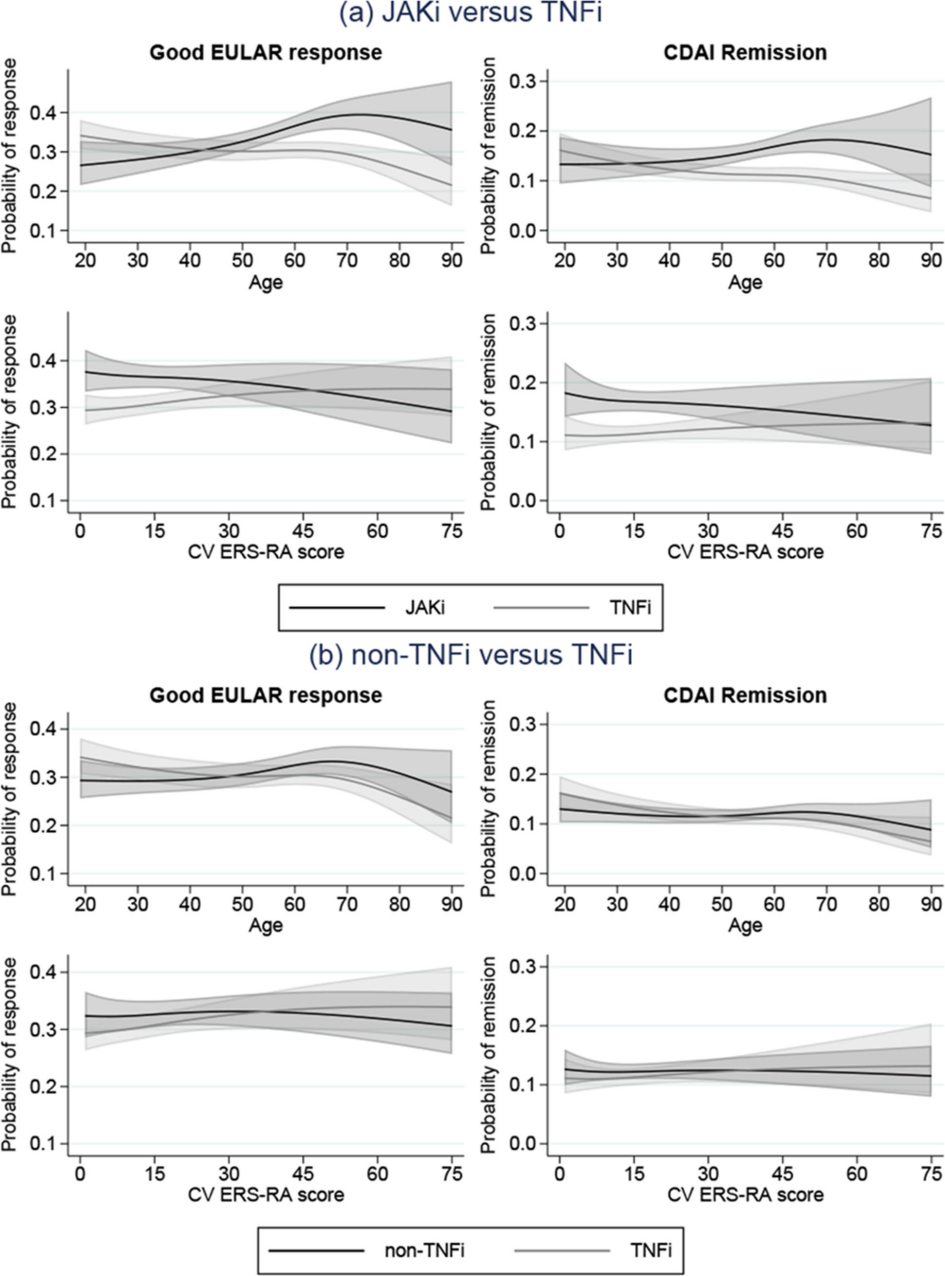
Predicted probability of response and remission at 6 months by age and CV risk of patients with RA treated with JAKi, non-TNFi and TNFi in Sweden. Presented for median covariate values (other than that on the X-axis), that is, the average RA individual; values available in online supplemental table S6. Estimated from Poisson regression models adjusting for baseline age, CV ERS-RA score, sex, smoking, DAS28 (response), CDAI (remission) year, seropositive RA, HAQ, VAS pain, RA duration, history of surgery, concomitant methotrexate and other csDMARD use, use of prednisolone in previous year, country of birth and education. csDMARD, conventional synthetic disease-modifying antirheumatic drug; CV, cardiovascular; ERS-RA, Expanded Risk Score in RA; HAQ, health assessment questionnaire; JAKi, janus kinase inhibitor; RA, rheumatoid arthritis; TNFi, tumour necrosis factor inhibitors.

**Table 1:** Proportion reaching EULAR good response and percentage point differences versus TNFi, by age, sex, presence of CV risk factors, and smoking

	Proportion (N) reaching outcome			Percentage point difference (95% CI) versus TNFi					
				Crude		Age, sex, and line of therapy adjusted		Fully adjusted*	
	JAKi n=*3166*	Non-TNFi n=*5575*	TNFi n=*11 286*	JAKi	Non-TNFi	JAKi	Non-TNFi	JAKi	Non-TNFi
Overall	34% (1074)	34% (1891)	38% (4228)	−3.5% (−6.2% to –0.9%)	−3.5% (−5.8% to –1.2%)	5.7% (2.4% to 9.0%)	3.9% (1.1% to 6.6%)	5.0% (2.2% to 7.9%)	2.5% (0.0% to 4.9%)
Sex									
Male	33% (189/577)	36% (431/1212)	41% (1046/2572)	−7.9% (−29.4% to 13.5%)	−5.2% (−22.4% to 12.1%)	1.2% (−3.7% to 6.1%)	1.4% (−2.5% to 5.4%)	0.5% (−4.0% to 5.0%)	−0.3% (−3.8% to 3.2%)
Female	34% (885/2589)	34% (1461/4363)	37% (3182/8714)	−2.3% (−12.9% to 8.3%)	−3.0% (−12.2% to 6.1%)	6.8% (4.2% to 9.4%)	4.6% (2.3% to 6.8%)	6.1% (4.0% to 8.2%)	3.3% (1.3% to 5.3%)
Age									
<65 years	33% (647/1945)	32% (972/3046)	38% (2745/7363)	−4.0% (−15.2% to 7.1%)	−5.4% (−16.3% to 5.5%)	5.7% (2.9% to 8.5%)	2.9% (0.4% to 5.5%)	4.4% (2.1% to 6.7%)	1.2% (−1.1% to 3.4%)
65+ years	35% (427/1221)	36% (920/2529)	38% (1483/3923)	−2.8% (−19.1% to 13.4%)	−1.5% (−16.0% to 13.1%)	5.6% (2.0% to 9.2%)	4.6% (1.4% to 7.9%)	5.9% (2.7% to 9.0%)	3.9% (1.0% to 6.7%)
CV risk factors									
No	27% (511/1871)	25% (764/3029)	33% (2383/7180)	−5.9% (−17.7% to 5.9%)	−8.0% (−18.4% to 2.4%)	3.9% (1.0% to 6.8%)	0.4% (−1.8% to 2.7%)	2.8% (0.4% to 5.3%)	−0.5% (−2.6% to 1.6%)
Yes	44% (563/1295)	44% (1128/2546)	45% (1845/4106)	−1.4% (−15.6% to 12.7%)	−0.6% (−16.8% to 15.5%)	7.9% (4.8% to 11.1%)	6.5% (2.8% to 10.2%)	8.3% (5.3% to 11.4%)	6.3% (3.1% to 9.5%)
Smoking									
Never smoker	35% (463/1327)	35% (771/2208)	39% (1926/4914)	−4.3% (−18.0% to 9.4%)	−4.3% (−16.8% to 8.3%)	5.0% (1.8% to 8.2%)	3.5% (0.8% to 6.3%)	3.7% (1.0% to 6.4%)	1.8% (−0.8% to 4.3%)
Ever smoker	33% (611/1839)	33% (1120/3367)	36% (2302/6372)	−2.9% (−15.8% to 10.0%)	−2.9% (−15.3% to 9.5%)	6.2% (3.1% to 9.3%)	4.2% (1.2% to 7.1%)	6.0% (3.3% to 8.7%)	2.9% (0.3% to 5.6%)

*Fully-adjusted risk differences estimated from linear regression models adjusting for sex, age, CV risk (Y/N), smoking, line of therapy, baseline DAS28CRP, seropositive RA, HAQ, VAS pain, RA duration, history of joint surgery, concomitant methotrexate and other csDMARD use, prednisolone use in previous year, year of treatment initiation, origin and education. Estimates presented by sex, age, CV risk and smoking include a cohort-sex, cohort-age, cohort-CV risk, cohort-smoking interaction in the model, respectively. Separate models fitted for JAKi versus TNFi, and non-TNFi versus TNFi. Positive percentage point difference values indicate that JAKi or TNFi have a higher proportion of response outcomes versus non-TNFi.

CV, cardiovascular; EULAR, European Alliance of Associations for Rheumatology; HAQ, health assessment questionnaire; JAKi, janus kinase inhibitor; RA, rheumatoid arthritis.

**Figure 2:**
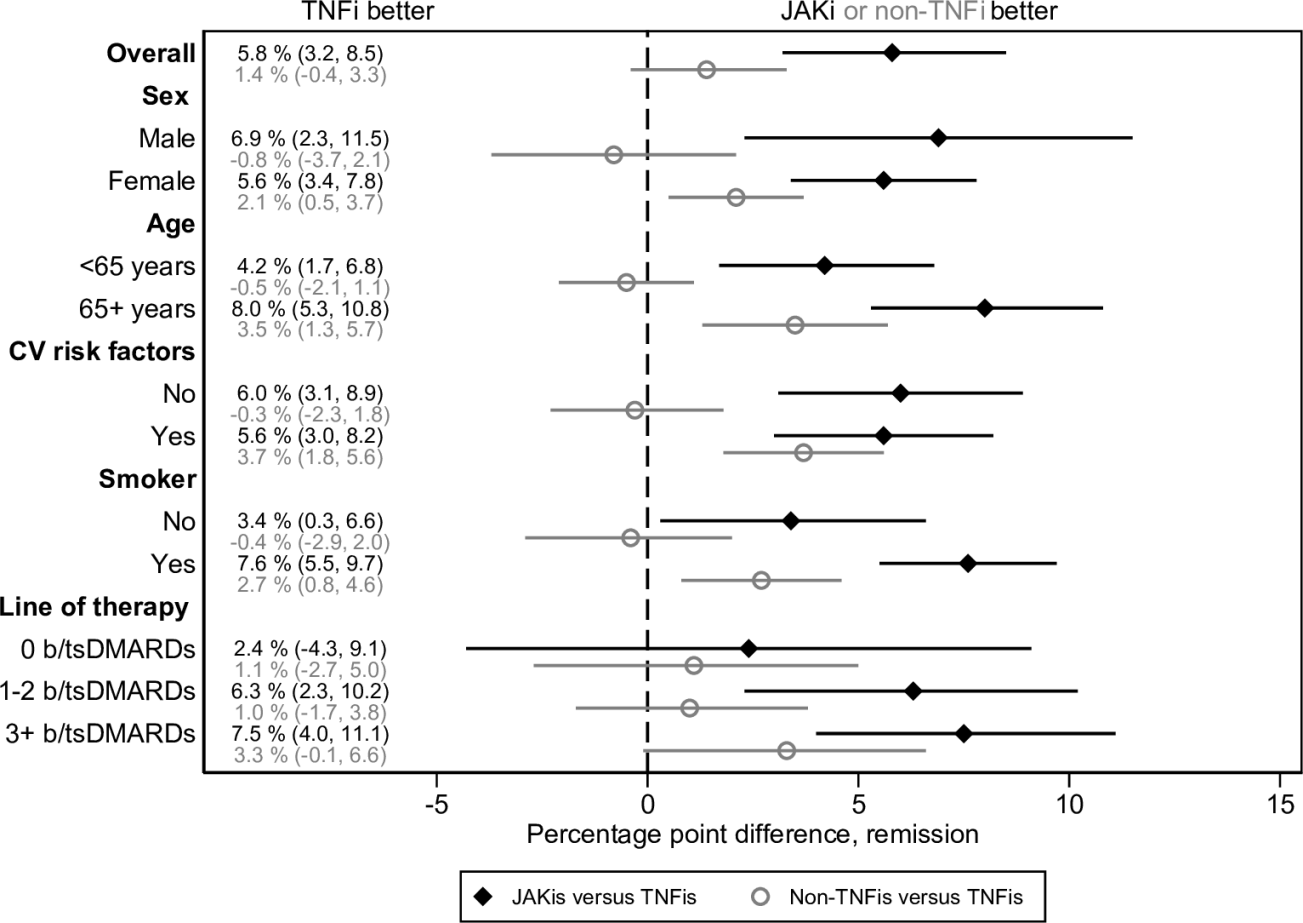
Percentage point differences for JAKis and non-TNFis (vs TNFis) of reaching CDAI remission at 6 months, presented overall, by sex, cardiovascular (CV) risk groups and line of therapy. b/tsDMARD, biological or targeted synthetic disease-modifying antirheumatic drug; JAKi, janus kinase inhibitor; TNFi, tumour necrosis factor inhibitors.

Overall, 502 (16%), 717 (13%) and 2293 (20%) of JAKi, non-TNFi and TNFi initiations reached CDAI remission at 6 months, see table 2. When adjusting for covariates, JAKi performed better with a percentage point difference of +5.8% (95% CI 3.2% to 8.5%) versus TNFi, and +1.4% (95% CI -0.4% to 3.3%) for non-TNFi of versus TNFi, see figure 2.

**Table 2:** Proportion with CDAI remission and percentage point difference versus TNFi, by age, sex, CV risk factor presence and smoking

	Proportion (N) reaching outcome			Percentage point difference (95% CI) versus TNFi					
				Crude		Age, sex, and line of therapy adjusted		Fully adjusted*	
	JAKi n=*3166*	Non-TNFi n=*5575*	TNFi n=*11 286*	JAKi	Non-TNFi	JAKi	Non-TNFi	JAKi	Non-TNFi
Overall	16% (502)	13% (717)	20% (2293)	−3.4% (−6.0% to –0.8%)	−5.9% (−7.9% to –3.8%)	4.9% (2.3% to 7.5%)	−0.7% (−2.5% to 1.2%)	5.8% (3.2% to 8.5%)	1.4% (−0.4% to 3.3%)
Sex									
Male	22% (125/577)	16% (188/1212)	25% (634/2572)	−1.4% (−25.6% to 22.7%)	−7.6% (−23.7% to 8.4%)	5.9% (1.4% to 10.4%)	−2.9% (−5.7% to –0.1%)	6.9% (2.3% to 11.5%)	−0.8% (−3.7% to 2.1%)
Female	15% (377/2589)	12% (529/4363)	19% (1660/8714)	−3.6% (−14.1% to 6.9%)	−5.3% (−13.7% to 3.1%)	4.6% (2.5% to 6.7%)	−0.0% (−1.7% to 1.6%)	5.6% (3.4% to 7.8%)	2.1% (0.5% to 3.7%)
Age									
<65 years	15% (298/1945)	12% (351/3046)	22% (1592/7363)	−5.5% (−17.5% to 6.5%)	−8.5% (-16.4% to –0.7%)	3.0% (0.5% to 5.4%)	−3.1% (−4.7% to –1.6%)	4.2% (1.7% to 6.8%)	−0.5% (−2.1% to 1.1%)
65+ years	17% (204/1221)	15% (366/2529)	18% (702/3923)	0.3% (−15.2% to 15.9%)	−1.7% (−14.3% to 11.0%)	7.0% (4.3% to 9.7%)	1.6% (−0.6% to 3.7%)	8.0% (5.3% to 10.8%)	3.5% (1.3% to 5.7%)
CV risk factors									
No	19% (347/1871)	13% (402/3029)	23% (1629/7180)	−3.7% (−17.2% to 9.7%)	−7.9% (−17.9% to 2.2%)	5.0% (2.2% to 7.9%)	−2.4% (−4.5% to –0.4%)	6.0% (3.1% to 8.9%)	−0.3% (−2.3% to 1.8%)
Yes	12% (155/1295)	12% (315/2546)	16% (664/4106)	−2.2% (−17.3% to 13.0%)	−2.1% (−15.7% to 11.4%)	4.9% (2.3% to 7.4%)	2.1% (0.2% to 4.0%)	5.6% (3.0% to 8.2%)	3.7% (1.8% to 5.6%)
Smoking									
Never smoker	15% (193/1327)	12% (260/2208)	22% (1078/4914)	−6.2% (−21.1% to 8.8%)	−8.7% (−20.9% to 3.5%)	2.1% (−1.0% to 5.2%)	−2.9% (−5.4% to –0.3%)	3.4% (0.3% to 6.6%)	−0.4% (−2.9% to 2.0%)
Ever smoker	17% (309/1839)	14% (457/3367)	19% (1215/6372)	−1.4% (−14.2% to 11.4%)	−3.8% (−14.5% to 6.9%)	6.9% (4.8% to 9.0%)	0.8% (−0.9% to 2.6%)	7.6% (5.5% to 9.7%)	2.7% (0.8% to 4.6%)

*Fully-adjusted risk differences estimated from linear regression models adjusting for sex, age, CV risk (Y/N) smoking, line of therapy, CDAI, seropositive RA, HAQ, VAS pain, RA duration, history of joint surgery, concomitant methotrexate and other csDMARD use, prednisolone use in previous year, year of treatment initiation, origin and education. Estimates presented by sex, age, CV risk and smoking include a cohort-sex, cohort-age, cohort-CV risk and cohort-smoking interaction in the model, respectively. Separate models fitted for JAKi versus TNFi, and non-TNFi versus TNFi. Positive percentage point difference values indicate that JAKi or TNFi have a higher proportion of response outcomes versus non-TNFi.

csDMARD, conventional synthetic disease-modifying antirheumatic drug; CV, cardiovascular; HAQ, health assessment questionnaire; JAKi, janus kinase inhibitor; RA, rheumatoid arthritis; TNFi, tumour necrosis factor inhibitors.

### Effectiveness by age, sex, CV risk groups and line of therapy

Within all sex, age, CV risk and smoking groups, the adjusted percentage point differences indicated a larger proportion reaching EULAR good response and CDAI remission for JAKi versus TNFi (see figures 1 and 2, and tables 1 and 2). Among these, the adjusted percentage point estimates were statistically different in those with (vs in those without) a CV risk factor (p=0.03) with respect to reaching EULAR good response at 6 months. For the other contrasts, the numerical differences in percentage point differences did not attain statistical significance, see online supplemental table S5 for all results.

Percentage point estimates within line of therapy groups indicated a benefit of JAKi versus TNFi only in those with one or more previous b/tsDMARD; see table 3 and figures 1 and 2.

**Table 3:** Proportion reaching EULAR good response and CDAI remission, respectively, and percentage point differences versus TNFi, by line of therapy

	Proportion (N) achieving outcome			Percentage point difference (95% CI) versus TNFi			
				Crude		Fully adjusted*	
	JAKi n=*3166*	Non-TNFi n=*5575*	TNFi n=*11 286*	JAKi	Non-TNFi	JAKi	Non-TNFi
EULAR good response							
0 b/tsDMARDs	7% (143/383)	45% (530/1176)	44% (3061/6968)	−6.6% (−28.9% to 15.6%)	1.1% (−12.1% to 14.4%)	−5.4% (−12.4% to 1.6%)	0.7% (−3.3% to 4.8%)
1–2 b/tsDMARDs	37% (450/1215)	33% (884/2661)	28% (1005/3593)	9.0% (−5.3% to 23.4%)	5.2% (−8.1% to 18.5%)	7.3% (3.2% to 11.4%)	2.7% (−1.0% to 6.4%)
≥3 b/tsDMARDs	31% (481/1568)	28% (477/1738)	22% (161/725)	8.5% (−6.7% to 23.6%)	5.2% (−11.0% to 21.5%)	8.4% (3.8% to 13.0%)	5.1% (0.4% to 9.8%)
CDAI remission							
0 b/tsDMARDs	26% (100/383)	23% (271/1176)	25% (1741/6968)	1.1% (−20.0% to 22.2%)	−2.0% (−13.7% to 9.8%)	2.4% (−4.3% to 9.1%)	1.1% (−2.7% to 5.0%)
1–2 b/tsDMARDs	19% (228/1215)	12% (330/2661)	15% (521/3593)	4.3% (−8.4% to 16.9%)	−2.1% (−10.8% to 6.5%)	6.3% (2.3% to 10.2%)	1.0% (−1.7% to 3.8%)
≥3 b/tsDMARDs	11% (175/1568)	7% (116/1738)	4% (31/725)	6.8% (−4.3% to 18.0%)	2.4% (−8.5% to 13.3%)	7.5% (4.0% to 11.1%)	3.3% (−0.1% to 6.6%)

*Fully-adjusted risk differences estimated from linear regression models adjusting for sex, age, CV risk (Y/N) smoking, baseline DAS28CRP (for response outcome) and baseline CDAI (for remission outcome) seropositive RA, HAQ, VAS pain, RA duration, history of joint surgery, concomitant methotrexate use, concomitant other csDMARD use prednisolone use in previous year, year of treatment initiation, origin, education, and an interaction between line of therapy and cohort. Separate models fitted for JAKi versus TNFi, and non-TNFi versus TNFi. Positive percentage point difference values indicate that JAKi or TNFi have a higher proportion of response outcomes versus non-TNFi.

b/tsDMARD, biological or targeted synthetic disease-modifying anti-rheumatic drug; csDMARD, conventional synthetic disease-modifying antirheumatic drug; CV, cardiovascular disease; EULAR, European Alliance of Associations for Rheumatology; HAQ, health assessment questionnaire; JAKi, janus kinase inhibitor; RA, rheumatoid arthritis; TNFi, tumour necrosis factor inhibitors.

### Effectiveness for continuous definitions of age and CV risk

Figure 3 presents predicted probabilities of EULAR good response and CDAI remission for each treatment response across age defined as a continuous variable and CV risk defined by the ERS-RA score, for a typical patient with RA (see online supplemental table S6 for covariate pattern). For both outcomes, the probability point estimates were generally higher for the JAKi cohort in comparison to both TNFi and non-TNFi. For this particular covariate pattern, the CIs for JAKi and TNFi predictions did not cross over the entire age (approximately 50–75 years) and ERS-RA score (approximately<20) scales. Inclusion of cohort-CV ERS-RA and cohort-age interactions were significant for age, and insignificant for ERS-RA score; p values for age and ERS-RA in the model using EULAR good response as outcome were 0.01 and 0.10, respectively, and 0.02 and 0.24 in models using CDAI remission.

For EULAR good response and CDAI remission, on the risk difference and risk ratio scale (online supplemental figures S1 and S2), adjusted estimates indicated a difference between response in JAKi versus TNFi, favouring JAKi, for those aged approximately over 50 years, and for those with a lower (approximately 30) CV ERS-RA score, for both response and remission.

### Sensitivity analyses

Results from complete case analyses, and results on the risk ratio (rather than risk difference) scale, were similar to those of the main analyses (online supplemental table S7-S9), as were results when excluding those with no previous b/tsDMARD (online supplemental figures S3 and S4). When restricting to individuals with only one prior TNFi use (online supplemental figures S5 and S6), results for JAKis versus TNFis were slightly more in favour of JAKis, and slightly less in favour of non-TNFi (for non-TNFi vs TNFi). Results when restricting to the period prior to the COVID pandemic drew similar conclusions to the main analyses (online supplemental figures S7 and S8).

## Discussion

In this Swedish population-based relative effectiveness study of patients with RA initiating JAKi, non-TNFi bDMARDs or TNFi in clinical practice, absolute numbers indicated that 20% of TNFi initiators reached remission at 6 months, versus 16% and 13% on JAKi and non-TNFi bDMARDs (38%, 34% and 34%, respectively, reaching EULAR good response), but the adjusted relative effectiveness of JAKi was overall greater than for TNFi (+5.0%, 95% CI 2.2% to 7.9% for EULAR good response, and +5.8%, 95% CI 3.2% to 8.5% for CDAI remission). For those individuals with (vs without) a safety risk factor (here: age, presence of CV risk factors, smoking), these percentage-unit differences were numerically even higher (with the exception of presence of CV risk factor in analyses with outcome remission), although not uniformly statistically significantly so. A similar but less pronounced pattern was seen when non-TNFi was contrasted to TNFi. When we investigated predicted probabilities of response and remission across continuous age and CV risk scores, instead of adjusted proportions based on categorised variables for specific covariate patterns, we noted certain patient segments (ages>50 years, with low CV risk scores) where the relative effectiveness of JAKi in comparison to TNFi was significantly elevated.

Our current results regarding the *overall* effectiveness with JAKis are in alignment with randomised control trials (RCTs),^12 14 22 23^ a multicentre non-randomised study,^24^ observational studies including a previous study from our group^10 25^ and a meta-analysis.^26^ Of course, overall comparisons and conclusions between RCTs and observational studies are difficult due to differences in design and inclusion criteria but generally lean towards the same conclusion: non-inferiority of JAKis versus bDMARDs,^27^ with potential signals that upadacitinib and baricitinib are more effective than other JAKis.^10 27^ To our knowledge, our study is the first observational study to assess the comparative effectiveness of JAKis versus TNFis within CV risk groups other than age. Our results showed non-statistically significant differences in relative effectiveness between those aged 65 and older, versus those younger than 65 years, a finding in alignment with other studies. A Japanese retrospective cohort study in patients with RA aged 65 and older found similar effectiveness of JAKis and bDMARDs at 24 weeks after treatment initiation, which was concluded to be comparable to results seen for younger patients from RCTs.^28^ Other analyses of phase III trials have found similar effectiveness of JAKis in elderly and younger patients with RA.^29 30^

We addressed our research question of interest in two different ways: first by selecting binary risk factors or subgroups, and second by considering the effect of continuous age and CV risk. The former attempted to provide a simpler guidance for treatment selection and alignment with the patient groups concerned by the warnings and precaution issued by regulatory agencies. It is not surprising that determining effectiveness within patient subgroups would be more complex than this binary selection of patients which led us to our second approach. We found a difference in the relative effectiveness of JAKis by binary CV risk for response, but on the continuous scale we saw JAKis had a better predicted response and remission for ERS-RA scores below approximately 20–30. This variation in results for CV risk should be interpreted in light of the following: (1) our elevated CV risk (binary) variable was selected to resemble the inclusion criteria in the ORAL surveillance trial and not necessarily to be in alignment with the ERS-RA score (note that sensitivity analyses using a ERS-RA cut-off at 7.5 found similar results, not shown here); (2) high ERS-RA scores are uncommon, the median value was around 15 despite the 0–100 scale, leading us to present results for values 0–75 on this scale; (3) the analytical approaches vary both in terms of modelling and interpretation of results, for example, the continuous approach is presented for a typical patient with RA and varies across other covariate patterns; and (4) dichotomised variables can generally hide effects that can be seen on a continuous scale.

Our study represents the treatment landscape in Sweden across the study period, by including all first treatment initiations from between 2016 and 2022. We accommodated channelling bias via both adjustment and stratification, but also performed sensitivity analyses to attempt to understand the impact of this via changes in the study design. We performed analyses with three different approaches, our main approach allowing for all first initiations per compound, and two sensitivity analyses focusing on (1) removal of previously b/tsDMARD-naïve patients, and (2) including only those with one prior TNFi use. While our main analyses were selected to include all b/tsDMARD initiations, irrespective of line of therapy to reflect treatment as used in clinical practice, it is important to note that the main analyses include a heterogeneous group of patients with RA. The small number of patients initiating JAKi as first ever b/tsDMARD may represent a selected group of patients, and because of their small number, the result of our main analysis is largely reflective of JAKi as second or later b/tsDMARD. Our two preplanned sensitivity analyses reflect different situations, with large differences in the b/tsDMARD experience of patients which arguably make the patients in the treatment cohorts more comparable. Despite these differences in design, results from these sensitivity analyses pointed towards largely similar patterns, save for precision. These analyses thus indicate that our main results are not critically dependent on the exact study design.

Further work would need to look into more depth to see if there remain other patient populations where the effectiveness of JAKis in RA is especially elevated in comparison to other bDMARDs, and to use this as part of a more comprehensive assessment of risks and benefits of JAKis to help aid clinicians in selecting the best treatment for patients with RA. Our study has some limitations. First, for some variables, we had non-negligible amounts of missing data. We attempted to reduce missing outcome information via using data on discontinuation, and via multiple imputation using chained equations for missing information on all variables. We also performed a complete case analysis as a sensitivity analysis which showed that conclusions were robust when using this approach. Second, despite our best attempts to adjust for confounders, there is a risk of residual confounding. Confounding can also create a pattern of effect modification, if different across levels of a variable. We noted a marked difference comparing JAKi (vs TNFi) in individuals who were previously b/tsDMARD-naïve versus those with previous b/tsDMARD exposure. In this case, and since JAKi in Sweden and during the study period are typically only used as first ever b/tsDMARD among patients with specific reasons not to use any of the available bDMARDs, the ‘discrepant’ results in this stratum may very well reflect residual confounding. It is also worth noting the high usage of steroids in our study population, which could inflate the absolute probabilities for remission; however, relative effects adjusted for steroid use should be unaffected by this. Third, the results reflect the Swedish setting, both in terms of patient characteristics and b/tsDMARD use in RA, and generalisability of results apply to similar populations initiating b/tsDMARDs. It is important to note that the majority of JAKi use in Sweden is baricitinib (here, 74% of all JAKi initiations); should different JAKi mechanisms affect effectiveness then our results would largely present that seen for JAK1 and JAK2 inhibition. We chose to study response and remission during the first months after treatment start, which are robust and clinically well-recognised outcomes. We cannot exclude that the observed contrasts remain also with longer-term outcomes. Finally, following the regulatory agencies’ expanded warnings and precautions regarding the safety profile of the class of JAKis, changes in prescription patterns may have occurred during the latter part of our study period 2016–2022, and will continue to do so in the future.

Our study also has some strengths worth noting. Our setting allowed us to perform a nationwide population-based study which covers almost all of the Swedish population, and the vast majority of all b/tsDMARD use in RA. Our registry linkage allowed us to adjust for a wide range of potential confounders and effect modifiers. We were also able to contextualise the relative effectiveness of JAKi versus TNFi by including also non-TNFis. We were able to assess elevated CV risk and age both as discrete and as continuous variables; the former being the better approach when considering a clinical application of results and selection of treatment, and the latter showing the potential complexity of the question at hand.

To conclude, the chances of primary response and remission with JAKis are overall higher than with TNFi as used in clinical practice, that is, mainly as second or later b/tsDMARD: among patients with risk factors of concern (age, smoking and CV risk), this effectiveness is similar or numerically further increased, and often higher than among patients with no risk factors. When treatment options and benefit-to-risk ratios are considered, effectiveness in specific patient segments should be considered in addition to safety.

## Data Availability

No data are available.
