# Supplementary material for: Are JAKis more effective among elderly patients with RA, smokers and those with higher cardiovascular risk? A comparative effectiveness study of b/tsDMARDs in Sweden
Source: RMD Open. 2023 Dec 26;9(4):e003648. doi: 10.1136/rmdopen-2023-003648 (PMC10753711; doi:10.1136/rmdopen-2023-003648)

Supplementary Tables

Table S1: Baseline covariate definitions

**Variable**	**Description and functional form where included in regression models**
Characteristics at start of follow-up	
Age	Age at cohort entry.<br>Either categorical (<65 years versus ≥65 years), or as a continuous covariate using restricted cubic splines with 3 degrees of freedom, as specified in the analysis.
Female	Indicator for sex of individual
DAS28	DAS28 CRP value selected from the visit recorded in SRQ closest to treatment initiation between -90 to +30 days the initiation date.<br>Included in regression models as continuous with both linear and squared term.
CDAI	CDAI value selected from the visit recorded in SRQ closest to treatment initiation between -90 to +30 days the initiation date.<br>Included in regression models as continuous with both linear and squared term.
HAQ	HAQ value selected from the visit recorded in SRQ closest to treatment initiation between -90 to +30 days the initiation date.<br>Included in regression models as continuous with both linear and squared term.
VAS pain	VAS pain value selected from the visit recorded in SRQ closest to treatment initiation between -90 to +30 days the initiation date.<br>Included in regression models as continuous with both linear and squared term.
Seropositive RA	Indicator for seropositive disease (versus seronegative/unknown). Calculated using rheumatoid factor and ACPA values recorded in the SRQ.
Smoking	Taken from the SRQ visit closest to treatment initiation with information on smoking available. Categorised as smoker (ever/current) versus never smoker.
Number of previous biologics	Calculated using all available b/tsDMARD (biological/targeted synthetic disease modifying anti rheumatic drugs) information from the SRQ. Categorized into 0, 1-2 and ≥3 for inclusion in statistical analyses.
Drug dispensations	
Methotrexate use (MTX)	Dispensation of ATC code L04AX03 within a -90 to +15-day window of the treatment initiation. Indicator variable (Y/N).
csDMARD use	Dispensation of a csDMARD (other than MTX, ATC codes= L04AX01, A07EC01, L04AD01, P01BA01, M01CB01, L04AA06, L01AA01, P01BA02, L04AA13, M01CB03) recorded within a -90 to +15-day window of the treatment initiation. Indicator variable (Y/N).

Steroid use	Dispensation of ATC code H02AB06 recorded in year prior to treatment initiation. Indicator variable (Y/N).
Antidiabetics	Dispensation of ATC codes A10A, A10B, A10XA01 recorded in year prior to treatment initiation. Indicator variable (Y/N).
Antihypertensives	Dispensation of ATC codes C02A, C03, C07A, C07F, C08, C09 recorded in year prior to treatment initiation. Indicator variable (Y/N).
Lipid lowering	Dispensation of ATC C10A, C10B recorded in year prior to treatment initiation. Indicator variable (Y/N).
Prednisolone treatment prior 1 year categorized	Daily prednisolone use categorized into 0, 1-5,6-10, ≥10 milligrams. Total milligrams of prednisolone dispensed during a 1 year look back recorded in the prescribed drug register (ATC H02AB06) calculated, then divided by 365.24.
Comorbidities	
History of joint surgery	History of joint surgery recorded in the 10 years prior to cohort entry. Defined as record in National Patient Register (inpatient and outpatient components, operational codes: NGB, NFB, NBB, NHB, NHC, NHE, NHF, NHG, 8423, 8424, 8426, 8419, 8437, 8436, 8420, 8421, 8422, 8400-8415).<br>Indicator variable (Y/N).
History of diabetes	History of diabetes recorded in the 10 years recorded prior to cohort entry. Defined as a record in the National Patient Register (inpatient and outpatient components, ICD10: E10-E14, O24). Indicator variable (Y/N).
History of hyperlipidemia	History of hyperlipidemia recorded in the 10 years recorded prior to cohort entry. Defined as a record in the National Patient Register (inpatient and outpatient components, ICD10: E78). Indicator variable (Y/N).
History of hypertension	History of hypertension recorded in the 10 years recorded prior to cohort entry. Defined as a record in the National Patient Register (inpatient and outpatient components, ICD10: I10-I15). Indicator variable (Y/N).
History of MI	History of myocardial infarction recorded in the 10 years recorded prior to cohort entry. Defined as a record in the National Patient Register (inpatient and outpatient components, ICD10: I21-I22). Indicator variable (Y/N).
History of stroke	History of stroke recorded in the 10 years recorded prior to cohort entry. Defined as record in National Patient Register (inpatient and outpatient components, ICD10: I60-I69).<br>Indicator variable (Y/N).
CV ERS-RA score	CV risk value from 0-100, ERS-RA score<br>Equation:<br>CV risk =(1-0.99395^exp(0.0343*I[age ≤44 years] +<br>0.0801*I[age ≥45 and age ≤49 years] +<br>1.2099*[age ≥50 and age ≤54 years] +<br>1.2977*[age ≥55 and age ≤59 years] +

	1.5922*[age ≥60 and age ≤64 years] +<br>2.0880*[age ≥65 and age ≤69 years]+<br>2.2187*[age ≥70 and age ≤74 years] +<br>2.7600*[age ≥75 years] +<br>0.4207*I[history of diabetes] +<br>0.3229* I[history of hyperlipidemia] +<br>0.2056* I[history of hypertension] +<br>0.8686* I[smoker]+ 0.5525* I[male] +<br>0.2776* I[CDAI > 10] +<br>0.1644* I[HAQ>0.55] +<br>0.4758* I[prednisolone use in previous year] +<br>0.3563* I[RA duration >10 years]))*100;<br><br>I= indicator function, =1 if true, =0 otherwise.<br>Diabetes, hyperlipidemia and hypertension defined as above.<br>CDAI, HAQ and RA duration obtained from SRQ.<br>Prednisolone use defined using dispensations in the prescribed drug register.<br>Included in regression models as a continuous term via restricted cubic splines with 3 degrees of freedom.
Elevated CV risk (Y/N)	Indicator variable for CV risk factor (Y/N) based on emulating the oral surveillance inclusion criteria.<br>To have the CV risk factor, the following conditions must be met:<br>At least one CV risk factor:<br>• Hypertension in the past 5 years (any diagnosis of I10-I15 NPR), or dispensation of an antihypertensive drug in past year (C02A, C03, C07A, C07F, C08, C09)<br>• Dispensation of a lipid-lowering drug in the past 183 days (ATC: C10A, C10B, PDR)<br>• Diabetes in the past 5 years (any diagnosis of E10-E14, NPR) or dispensation of an antidiabetic drug in past year (ATC codes A10A, A10B, A10XA01)<br>• CVD in the past 5 years (main diagnosis of I20-I25, NPR)<br>• Family history of CVD ever (For female first-degree relatives: I20-I25 in NPR at age 65 or younger. For male first-degree relatives: I20-I25 at age 55 or younger) Relatives were identified using the Swedish Multigeneration Register and diagnoses identified from the National Patient Register (NPR).<br>• Smoker (ever or current smoker)<br>Plus the requirement of:<br>1. CRP >=3<br>2. DAS28CRP >=2.9<br>Both CRP and DAS28CRP values selected at the SRQ rheumatology visit closest to treatment initiation, between -90 to +30 days.
Socioeconomics	
Education	Highest education achieved as recorded in the year prior to cohort entry.<br>Data obtained from the Longitudinal integrated database for health

	insurance and labor market studies (LISA). Categorized into <12 years or ≥12 years.
Sweden born	Indicator for if the individual was born in Sweden or elsewhere.

Table S2: Number of treatment initiations imputed based on discontinuation within 6 months after treatment initiation

	**JAKi** N=3166	**Non-TNFi** N=5575	**TNFi** N=11 286
Non-responders/remission*	469/380 (15%/12%)	740/596 (13%/11%)	1432/1145 (13%/10%)
Responders/remission**	0/0 (0%/0%)	5/9 (0.1%/0.3%)	3/6 (0.03%/0.05%)

*Non-responders/remission imputed for those discontinuing treatment due to any reason except pregnancy and inactive disease
**Responder/remission imputation performed where individuals discontinued due to inactive disease

Table S3: Baseline characteristics of RA patients initiating JAKis, non-TNFis and TNFis in Sweden, non-imputed dataset

	JAKi	NonTNFi	TNFi
Treatment initiations	3166	5575	11 286
Individuals	2794	4700	9470
Age, median (IQR)	60 (51-70)	63 (52-71)	59 (48-69)
Female	82%	78%	77%
Disease-related factors			
RA duration years, median (IQR)	13.5 (7.1-22.7)	12.0 (5.4-21.2)	7.4 (2.7-15.4)
Missing duration, %	1%	1%	0%
Year of initiation, %	2019	2018	2019
Seropositive RA, %	78%	83%	77%
DAS28CRP, median (IQR)	4.3 (3.5-5.0)	4.4 (3.6-5.2)	4.1 (3.3-4.8)
DAS28CRP missing, %	41%	42%	39%
CDAI, median (IQR)	20.0 (14.0-27.5)	21.0 (14.5-28.5)	18.0 (12.5-25.5)
CDAI missing, %	43%	46%	43%
HAQ median (IQR)	1.1 (0.6-1.6)	1.1 (0.8-1.6)	0.9 (0.5-1.4)
HAQ missing, %	42%	42%	38%
VAS pain median (IQR)	62 (42-76)	63 (42-78)	56 (34-73)
VAS pain missing, %	36%	38%	34%
Smoking (ever/current), %	59%	61%	57%
Smoking missing, %	10%	13%	15%
Number of previous b/tsDMARDs, %			
0	12%	21%	62%
1-2	38%	48%	32%
3+	50%	31%	6%
Drug dispensations, %			
MTX use	31.7%	36.4%	52.9%
csDMARD use	11.2%	13.6%	18.3%
Steroid use	72.7%	75.6%	69%
Antidiabetics use	9.5%	10.8%	8.9%
Antihypertensive use	45.9%	47.6%	38%
Lipid lowering drug use	0.9%	1.3%	0.7%
Daily prednisolone use in prev year*	2.7 (0.0-5.5)	2.7 (0.0-5.5)	1.4 (0.0-4.2)
Comorbid conditions, %			
Joint surgery	5.8%	4.9%	3.7%
Diabetes	7.9%	8%	6%
Hyperlipidemia	3.6%	3.7%	2.7%
Hypertension	19.4%	18.7%	12.8%
MI	1.3%	1.5%	0.8%
Stroke	0.9%	1.1%	0.7%
CV ERS-RA score, median (IQR)	14.0 (5.2-28.3)	15.9 (5.4-29.9)	11.4 (3.7-23.1)
Missing ERS-RA score	54%	55%	54%

CV risk (Y/N)	42%	45%	37%
CV risk (Y/N) missing	41%	42%	39%
Socioeconomic factors, %			
Education >12 years	34%	31%	34%
Born in Sweden	86%	87%	87%
*Average daily dose (mg) calculated as the total prednisolone dispensations in the year prior to cohort entry, divided by 365			

**Table S4:** Baseline characteristics of RA patients initiating JAKis, non-TNFis and TNFis in Sweden, imputed dataset, imputed variables only			
	**JAKi**	**NonTNFi**	**TNFi**
Disease-related factors			
RA duration years, median (IQR)	13.5 (7.1-22.7)	12.0 (5.4-21.2)	7.4 (2.7-15.4)
DAS28CRP, median (IQR)	4.2 (3.4-5.0)	4.3 (3.6-5.1)	4.0 (3.2-4.8)
CDAI, median (IQR)	20.0 (14.0-27.0)	20.5 (14.5-28.0)	18.0 (12.5-25.0)
HAQ, median (IQR)	1.1 (0.8-1.6)	1.1 (0.8-1.6)	0.9 (0.5-1.4)
VAS pain, median (IQR)	61 (40-76)	62 (40-78)	55 (32-73)
Smoking (ever/current), %	58%	60%	56%
Comorbid conditions			
CV ERS-RA score, median (IQR)	14.6 (5.4-29.0)	16.4 (6.5-34.1)	11.4 (3.7-23.5)
CV risk (Y/N), %	41%	46%	36%
Socioeconomic factors			
Education >12 years, %	34%	31%	34%

Table S5: P-values for the inclusion of effect-modifying interaction terms from linear regression models.

Variable-cohort interaction model	Response		Remission	
	JAKi v TNFi	Non-TNFi v TNFi	JAKi v TNFi	Non-TNFi v TNFi
Sex	0.075	0.147	0.677	0.189
Age	0.551	0.215	0.094	**0.021**
CV risk (Y/N)	**0.030**	**<0.01**	0.868	**0.032**
Smoking	0.353	0.602	0.058	0.124
Line of therapy	**<0.01**	0.428	0.430	0.564

Table S6: Covariates values used for predicting probability of outcome

**Covariate**	**Value used for predictions**
Sex	Female
Age*	60 years
RA duration	<10 years
Year of initiation	2019
Seropositive RA	Seropositive RA
DAS28CRP	4.15
CDAI	19
HAQ	1
VAS pain	59
Smoker	Yes
Number of previous b/tsDMARDs	1-2
Concomitant csDMARD use	No
Concomitant methotrexate use	No
Prednisolone use in previous year	Average daily dose category with values 0.01-4.99
History of surgery	No
CV ERS-RA score*	12.6
Born in Sweden	Yes
Education >12 years	No

*predicted value used for results that are not presented over this variable continuously

Table S7: Adjusted risk ratios comparing EULAR good response and CDAI remission in JAKi and non-TNFi (verus TNFi)

	**Fully-adjusted* risk ratios (95% CI) versus TNFi**			
	**EULAR good response**		**CDAI remission**	
	JAKi	Non-TNFi	JAKi	Non-TNFi
**Overall**	1.17 (1.07, 1.28)	1.06 (0.99, 1.14)	1.42 (1.22, 1.65)	1.10 (0.97, 1.24)
**Sex**				
Male	1.02 (0.89, 1.16)	0.98 (0.88, 1.08)	1.54 (1.24, 1.90)	1.03 (0.88, 1.19)
Female	1.21 (1.13, 1.29)	1.09 (1.03, 1.16)	1.38 (1.21, 1.58)	1.13 (1.00, 1.27)
**Age**				
<65 years	1.16 (1.07, 1.25)	1.03 (0.96, 1.10)	1.32 (1.14, 1.52)	0.97 (0.87, 1.09)
65+ years	1.18 (1.07, 1.30)	1.09 (1.01, 1.18)	1.56 (1.33, 1.84)	1.23 (1.06, 1.43)
**CV risk**				
no CV risk	1.08 (0.99, 1.17)	0.94 (0.87, 1.02)	1.46 (1.26, 1.70)	1.03 (0.90, 1.17)
CV risk	1.27 (1.17, 1.37)	1.18 (1.09, 1.27)	1.33 (1.09, 1.61)	1.22 (1.07, 1.40)
**Smoking**				
Never smoker	1.13 (1.04, 1.24)	1.05 (0.98, 1.13)	1.24 (1.02, 1.50)	0.99 (0.83, 1.18)
Ever/current smoker	1.19 (1.10, 1.29)	1.07 (0.99, 1.16)	1.57 (1.39, 1.76)	1.18 (1.04, 1.33)

*Fully-adjusted risk ratios estimated from Poisson regression models adjusting for sex, age, CV risk (Y/N), plus line of therapy, baseline DAS28CRP (for response outcome) and baseline CDAI (for remission outcome) seropositive RA, HAQ, VAS pain, RA duration, history of joint surgery, concomitant csDMARD use, concomitant methotrexate use, prednisolone use in previous year, year of treatment initiation, origin and education. Estimates presented by sex, age, CV risk and smoking include a cohort-sex, cohort-age, cohort-CV risk and cohort-smoking interaction in the model, respectively. Separate models fitted for JAKi versus TNFi, and non-TNFi versus TNFi.

Table S8: Proportion reaching EULAR good response at 6 months and percentage point differences versus TNFi, by age, sex, CV risk factor presence and smoking, complete case

	**Proportion (N) achieving outcome**			**Percentage point difference (95% CI) versus TNFi**			
				**Crude**		**Fully-adjusted***	
	JAKi *N=1434*	Non-TNFi *N=2374*	TNFi *N=5009*	JAKi	Non-TNFi	JAKi	Non-TNFi
**Overall**	12.3% (212)	10.0% (285)	16.5% (988)	-4.2% (-6.0, -2.3)	-6.5% (-7.9, -5.0)	7.0% (4.1, 9.9)	4.7% (2.3, 7.0)
**Sex**							
Male	16.8% (51/303)	12.5% (76/606)	20.1% (267/1326)	-3.3% (-8.0, 1.4)	-7.6% (-11.0, -4.2)	1.8% (-4.3, 8.0)	2.1% (-2.7, 6.9)
Female	11.4% (161/1418)	9.3% (209/2247)	15.4% (721/4671)	-4.1% (-6.0, -2.1)	-6.1% (-7.7, -4.5)	8.2% (5.0, 11.4)	5.3% (2.8, 7.9)
**Age**							
<65 years	11.8% (124/1053)	9.1% (152/1673)	17.5% (683/3899)	-5.7% (-8.0, -3.5)	-8.4% (-10.3, -6.6)	7.8% (4.3, 11.3)	5.0% (2.2, 7.9)
65+ years	13.2% (88/668)	11.3% (133/1180)	14.5% (305/2098)	-1.4% (-4.3, 1.6)	-3.3% (-5.6, -0.9)	5.7% (1.3, 10.2)	3.8% (0.3, 7.4)
**CV risk**							
no CV risk	15.3% (97/634)	11.1% (115/1036)	18.4% (454/2470)	-3.1% (-6.3, 0.1)	-7.3% (-9.7, -4.8)	6.3% (2.1, 10.5)	2.7% (-0.6, 6.0)
CV risk	10.6% (51/479)	10.8% (91/842)	14.4% (232/1606)	-3.8% (-7.1, -0.5)	-3.6% (-6.4, -0.9)	9.5% (4.4, 14.6)	6.9% (2.9, 11.0)
**Smoking**							
Never smoker	11.6% (77/665)	9.3% (95/1023)	18.0% (403/2239)	-6.4% (-9.3, -3.5)	-8.7% (-11.1, -6.3)	6.6% (2.2, 11.0)	5.2% (1.5, 8.9)
Ever/current smoker	12.5% (116/926)	10.5% (160/1527)	15.4% (476/3083)	-2.9% (-5.4, -0.4)	-5.0% (-7.0, -3.0)	8.0% (4.2, 11.8)	5.0% (2.0, 8.0)

*Fully-adjusted risk differences estimated from linear regression models adjusting for sex, age, CV risk (Y/N), smoking, line of therapy, baseline DAS28CRP (for response outcome) and baseline CDAI (for remission outcome) seropositive RA, HAQ, VAS pain, RA duration, history of joint surgery, concomitant csDMARD use, concomitant methotrexate use, prednisolone use in previous year, year of

treatment initiation, origin and education. Estimates presented by sex, age, CV risk and smoking include a cohort-sex, cohort-age, cohort-CV risk and cohort-smoking interaction in the model, respectively. The Interaction terms for sex for JAKis versus TNFi was of borderline significance (p=0.058), all others were insignificant. Separate models fitted for JAKi versus TNFi, and non-TNFi versus TNFi. Positive values indicate that JAKi or TNFi have a higher proportion of response outcomes versus non-TNFi.

Table S9: Proportion reaching CDAI remission at 6 months and percentage point difference versus TNFi, by age, sex, CV risk factor presence and smoking, complete case

	Proportion (N) achieving outcome			Percentage point difference (95% CI) versus TNFi			
				Crude		Fully-adjusted*	
	JAKi *N=1721*	Non-TNFi *N=2853*	TNFi *N=5997*	JAKi	Non-TNFi	JAKi	Non-TNFi
**Overall**	12.3% (212)	10.0% (285)	16.5% (988)	-4.2% (-6.0, -2.3)	-6.5% (-7.9, -5.0)	4.6% (2.6, 6.7)	0.4% (-1.1, 2.0)
**Sex**							
Male	16.8% (51/303)	12.5% (76/606)	20.1% (267/1326)	-3.3% (-8.0, 1.4)	-7.6% (-11.0, -4.2)	4.8% (-0.1, 9.6)	-1.3% (-4.7, 2.2)
Female	11.4% (161/1418)	9.3% (209/2247)	15.4% (721/4671)	-4.1% (-6.0, -2.1)	-6.1% (-7.7, -4.5)	4.6% (2.4, 6.8)	0.9% (-0.8, 2.6)
**Age**							
<65 years	11.8% (124/1053)	9.1% (152/1673)	17.5% (683/3899)	-5.7% (-8.0, -3.5)	-8.4% (-10.3, -6.6)	3.1% (0.6, 5.6)	-1.3% (-3.2, 0.6)
65+ years	13.2% (88/668)	11.3% (133/1180)	14.5% (305/2098)	-1.4% (-4.3, 1.6)	-3.3% (-5.6, -0.9)	6.6% (3.5, 9.8)	2.2% (-0.2, 4.6)
**CV risk**							
no CV risk	14.5% (107/739)	11.1% (137/1229)	18.2% (525/2879)	-3.8% (-6.7, -0.9)	-7.1% (-9.3, -4.8)	5.6% (2.2, 9.1)	0.3% (-2.3, 2.9)
CV risk	11.0% (41/374)	10.6% (69/649)	13.5% (161/1197)	-2.5% (-6.2, 1.2)	-2.8% (-5.9, 0.2)	4.4% (1.0, 7.9)	3.1% (0.3, 6.0)
**Smoking**							
Never smoker	11.6% (77/665)	9.3% (95/1023)	18.0% (403/2239)	-6.4% (-9.3, -3.5)	-8.7% (-11.1, -6.3)	2.7% (-0.4, 5.8)	-1.1% (-3.6, 1.4)
Ever/current smoker	6.4% (10/156)	9.1% (25/275)	12.1% (75/619)	-5.7% (-10.3, -1.1)	-3.0% (-7.3, 1.2)	5.6% (2.9, 8.3)	1.6% (-0.5, 3.7)

*Fully-adjusted risk differences estimated from linear regression models adjusting for sex, age, CV risk (Y/N), smoking, line of therapy, baseline DAS28CRP (for response outcome) and baseline CDAI (for remission outcome) seropositive RA, HAQ, VAS

pain, RA duration, history of joint surgery, concomitant csDMARD use, concomitant methotrexate use, prednisolone use in previous year, year of treatment initiation, origin and education. Estimates presented by sex, age, CV risk and smoking include a cohort-sex, cohort-age, cohort-CV risk and cohort-smoking interaction in the model, respectively. Interaction terms for age (JAKi vs TNFi, p=0.06; non-TNFi vs TNFi, p=0.019) and smoker (non-TNFi vs TNFi, p=0.087) were borderline or statistically significant. None of the interaction terms were statistically significant (Wald tests p>0.05). Separate models fitted for JAKi versus TNFi, and non-TNFi versus TNFi.
Positive values indicate that JAKi or TNFi have a higher proportion of response outcomes versus non-TNFi.

Supplementary Figures

Figure S1: Predicted risk differences of response and remission at 6 months by age and CV ERS-RA score of patients with RA treated with JAKi and non-TNFi (versus TNFi) in Sweden. Estimated from linear regression models adjusting for baseline age, CV ERS-RA score, sex, smoking, line of therapy DAS28 (response), CDAI (remission) year, seropositive RA, HAQ, VAS pain, RA duration, history of surgery, concomitant csDMARD and methotrexate use, use of prednisolone in previous year, country of birth and education.

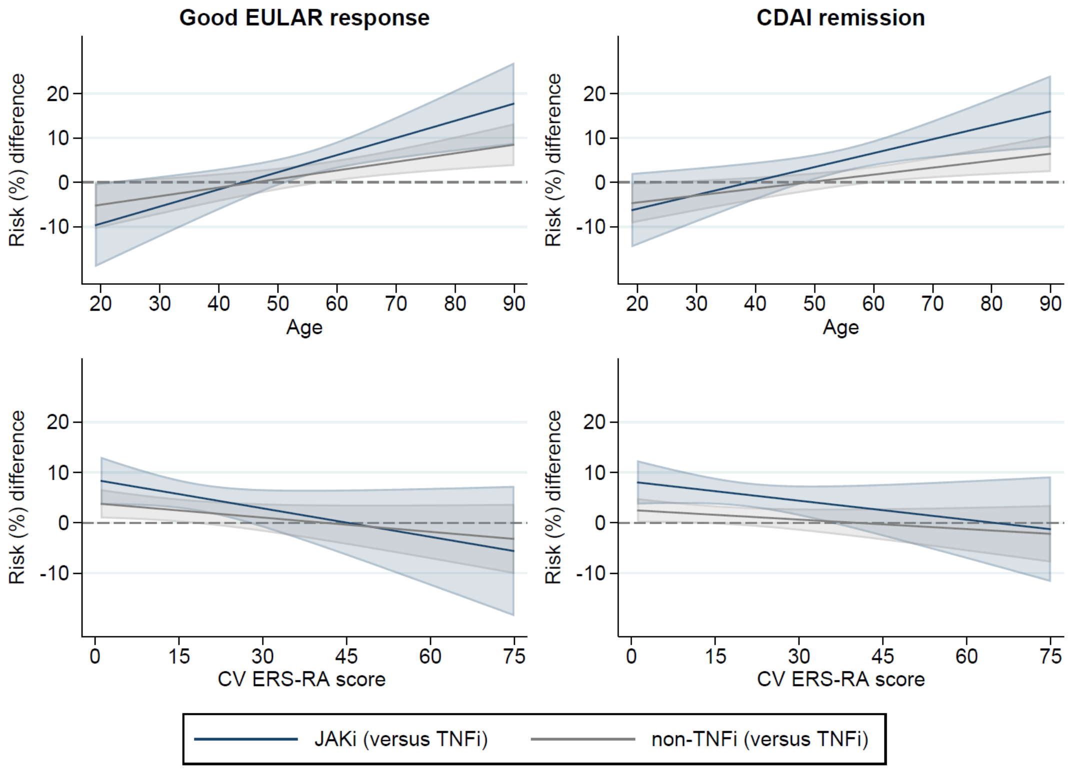

Figure S2: Predicted risk ratios of response and remission at 6 months by age and CV risk of patients with RA treated with JAKi and non-TNFi TNFi (versus TNFi) in Sweden. Estimated from Poisson regression models adjusting for baseline age, CV ERS-RA score, sex, smoking, line of therapy, DAS28 (response), CDAI (remission) year, seropositive RA, HAQ, VAS pain, RA duration, history of surgery, concomitant csDMARD and methotrexate use, use of prednisolone in previous year, country of birth and education.

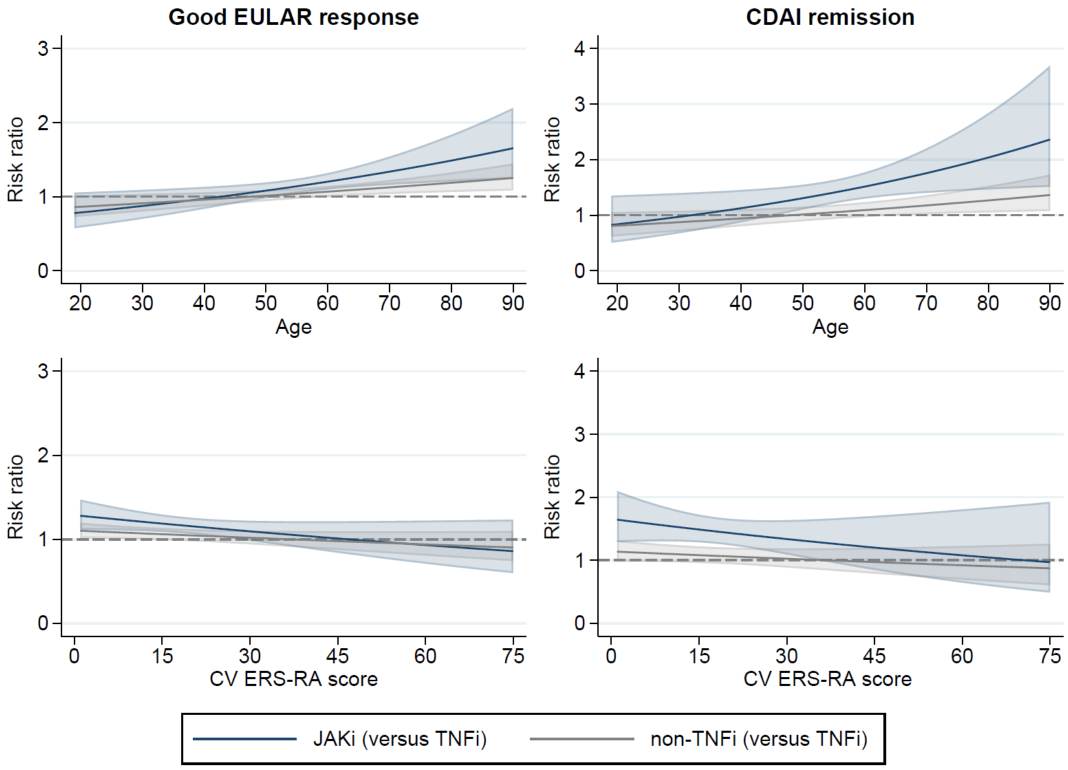

Figure S3: Percentage point differences for JAKis and non-TNFis (versus TNFis) of reaching EULAR good response at 6 months, presented overall, by sex, cardiovascular risk groups, and line of therapy; sensitivity analysis excluding those with no prior b/tsDMARD use

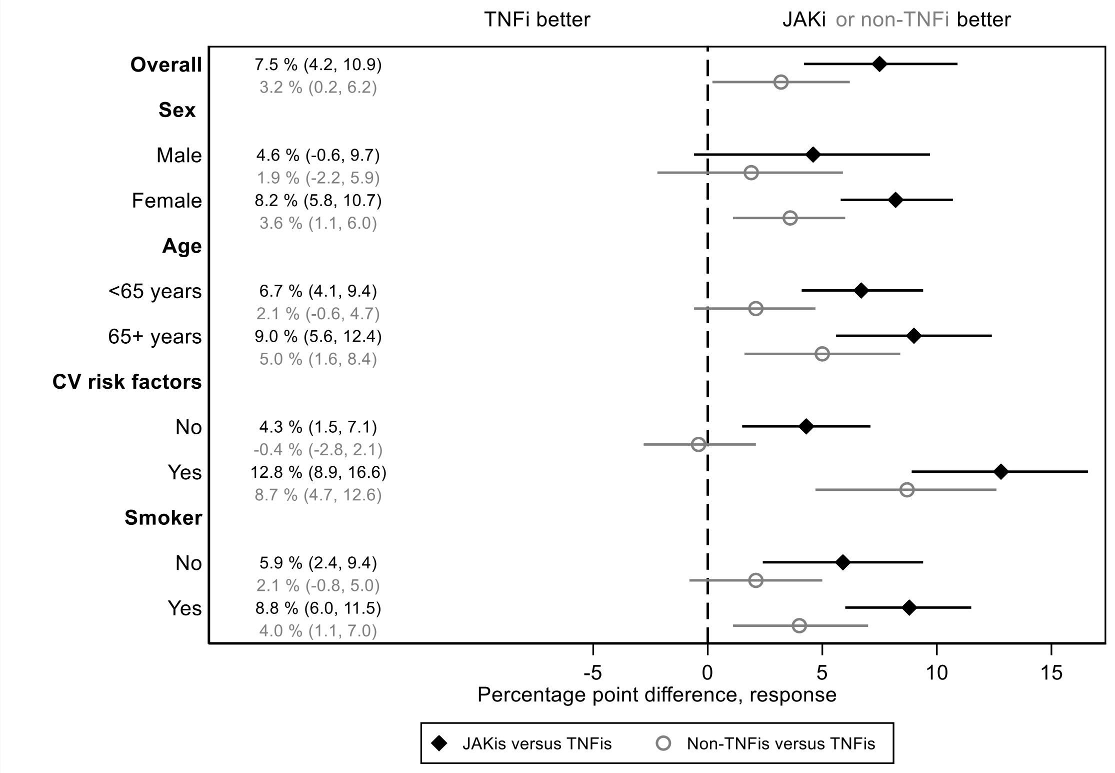

Figure S4: Percentage point differences of JAKis and non-TNFis (versus TNFi) of reaching CDAI remission at 6 months, presented overall, by sex, cardiovascular risk groups, and line of therapy; sensitivity analysis excluding those with no prior b/tsDMARD use

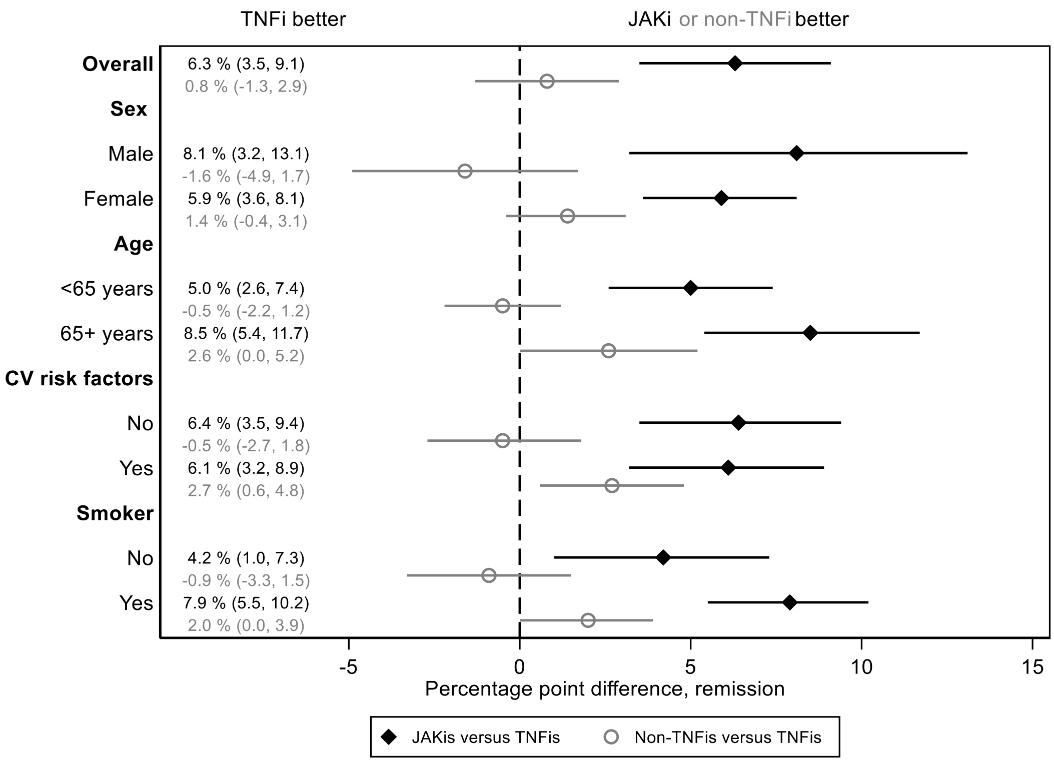

Figure S5: Percentage point differences for JAKis and non-TNFis (versus TNFis) of reaching EULAR good response at 6 months, presented overall, by sex, and cardiovascular risk groups; sensitivity analysis including only treatment initiations with only one prior TNFi use

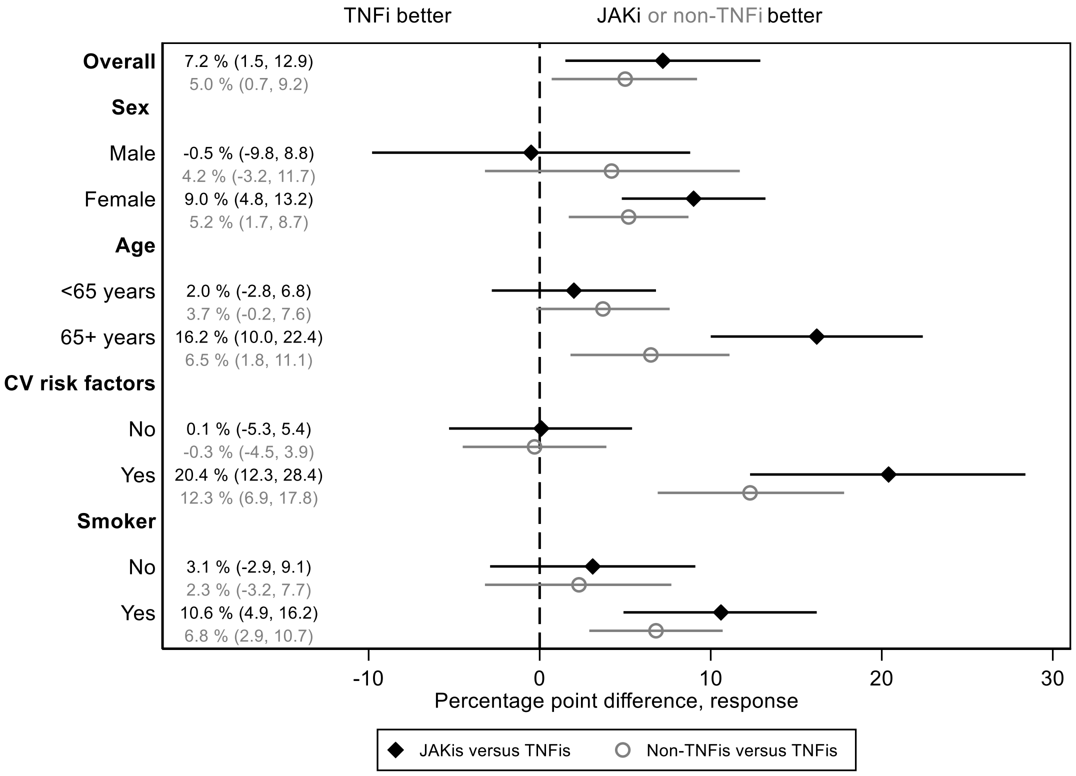

Figure S6: Percentage point differences of JAKis and non-TNFis (versus TNFi) of reaching CDAI remission at 6 months, presented overall, by sex and cardiovascular risk groups; sensitivity analysis including only treatment initiations with only one prior TNFi use

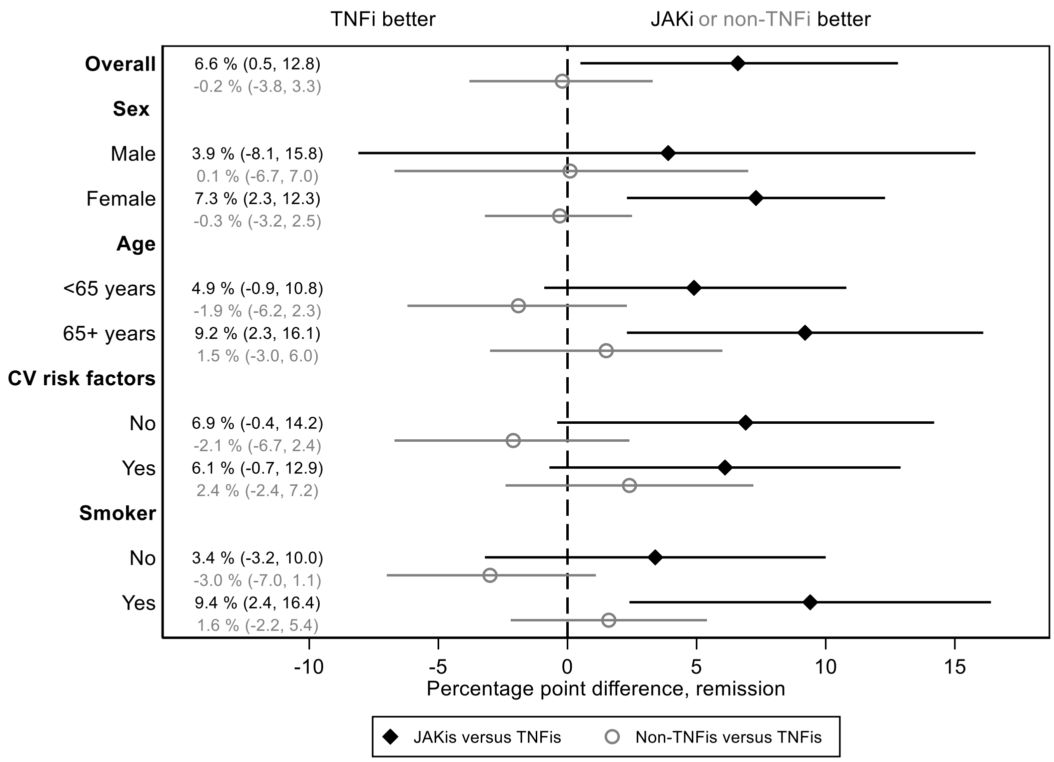

Figure S7: Percentage point differences of JAKis and non-TNFis (versus TNFi) of reaching EULAR good response at 6 months, presented overall, by sex and cardiovascular risk groups; sensitivity analysis excluding treatment initiations during or after the covid pandemic

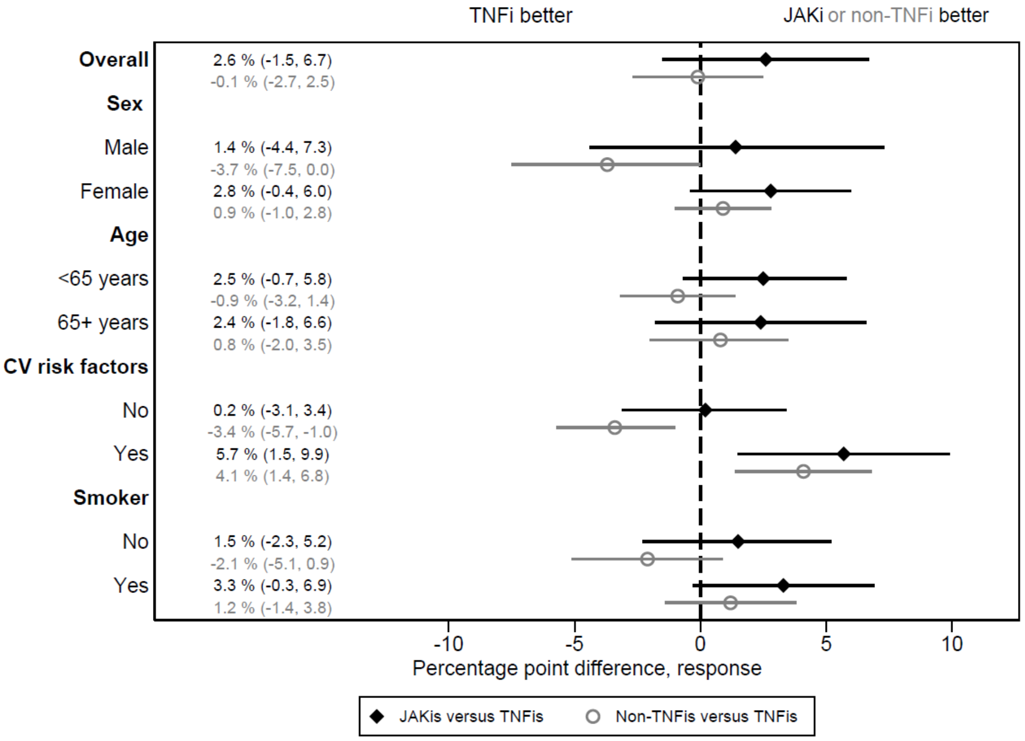

Figure S8: Percentage point differences of JAKis and non-TNFis (versus TNFi) of reaching CDAI remission at 6 months, presented overall, by sex and cardiovascular risk groups; sensitivity analysis excluding treatment initiations during or after the covid pandemic

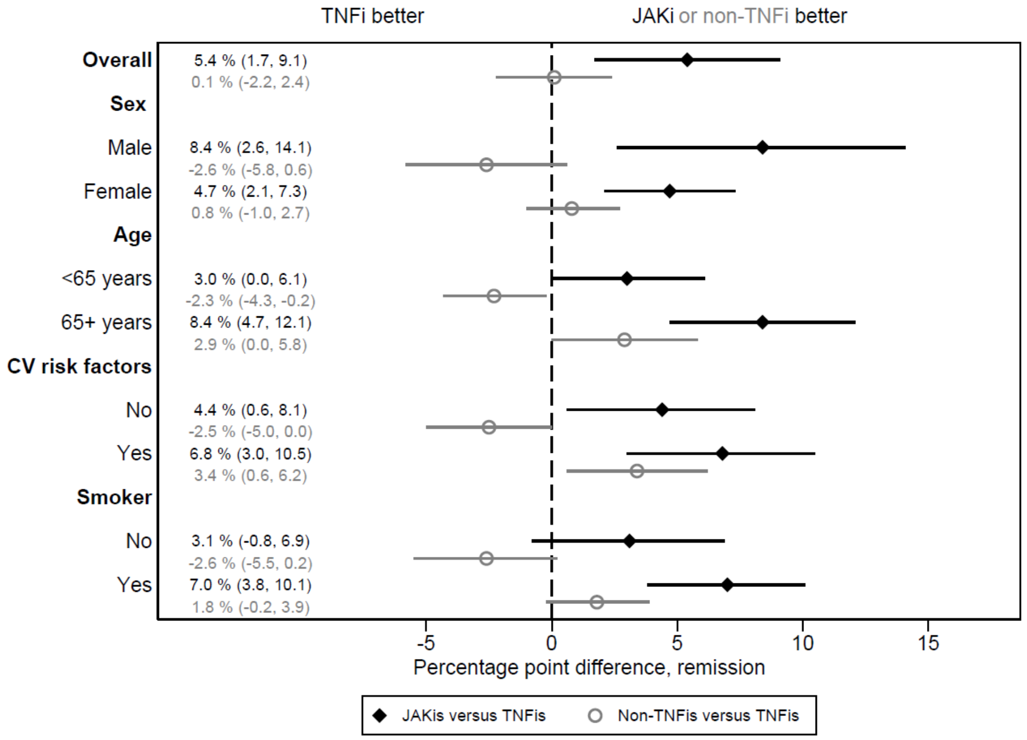

Supplement: Supplementary data [file rmdopen-2023-003648supp001.pdf]
